# Two-Way Detection of COVID-19 Spike Protein and Antibody Using All-Dielectric Metasurface Fluorescence Sensors

**DOI:** 10.3390/bios12110981

**Published:** 2022-11-07

**Authors:** Masanobu Iwanaga, Wanida Tangkawsakul

**Affiliations:** Research Center of Functional Materials, National Institute for Materials Science (NIMS), 1-1 Namiki, Tsukuba 305-0044, Japan

**Keywords:** COVID-19, SARS-CoV-2, spike glycoprotein, peptide, antibody, all-dielectric metasurface, fluorescence sensor, sandwich assay

## Abstract

COVID-19 (or SARS-CoV-2) has deeply affected human beings worldwide for over two years, and its flexible mutations indicate the unlikeliness of its termination in a short time. Therefore, it is important to develop a quantitative platform for direct COVID-19 detection and human status monitoring. Such a platform should be simpler than nucleic acid amplification techniques such as polymerase chain reaction, and more reliable than the disposable test kits that are based on immunochromatography. To fulfill these requirements, we conducted proof-of-concept experiments for the quantitative detection of spike glycoprotein peptides and antibodies in one platform, i.e., all-dielectric metasurface fluorescence (FL) sensors. The high capability to enhance FL intensity enabled us to quantitatively measure the glycoproteins and antibodies more efficiently compared with the previous methods reported to date. Furthermore, the intrinsic limit of detection in the metasurface FL sensors was examined via confocal microscopy and found to be less than 0.64 pg/mL for glycoprotein peptides. Moreover, the sensors had a dynamic range more than five orders that of the target concentrations, indicating extremely high sensitivity. These two-way functions of the metasurface FL sensors can be helpful in reducing daily loads in clinics and in providing quantitative test values for proper diagnosis and cures.

## 1. Introduction

For more than two years, the COVID-19 (SARS-CoV-2) pandemic has substantially reduced social and economic activities. To overcome such damages, efficient and quantitative techniques for evaluating the status of human bodies are required. Particularly, quantification of the concentrations of COVID-19 and neutralizing antibodies (Abs) are highly preferred for proper therapeutic approaches. Currently, polymerase chain reaction (PCR) [1] serves as a high sensitivity method for COVID-19 detection; however, it is widely known that PCR is costly as a daily practice and has high demand with regard to human resources. As handy chips, antigen test kits relying on paper-based immunochromatography are used; they are known to be far less sensitive than PCR and qualitative platforms for supplementary tests. Numerous proposals for detecting COVID-19 have been reported in the last two years, and are summarized in several review papers [2,3,4,5,6,7,8]. However, to the best of our knowledge, there is no newly established technique that meets the aforementioned criterion, suggesting practical difficulties that have not been noted in previous reports. Thus, there is room to explore a practical and quantitative technique to detect the antigens and Abs.

**Figure 1:**
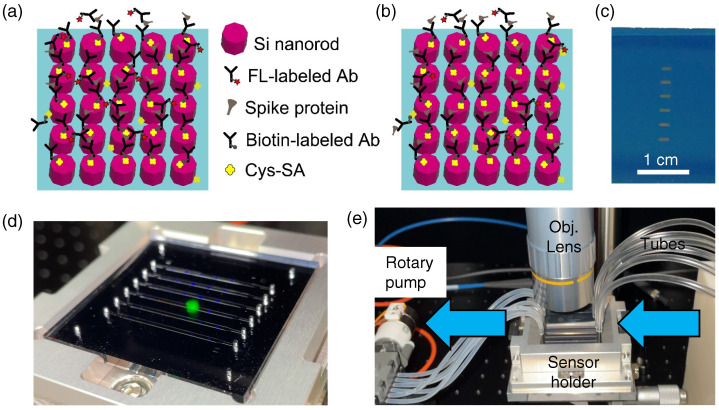
Schematics and overview of this study. Illustrations for detection of (**a**) glycoproteins and (**b**) antibodies (Abs) on the all-dielectric metasufaces of the Si nanorod array, which is located on the SiO${}_{2}$ layer (pale blue). Si nanorods, labeled Abs, spike protein, and Cys-SA are listed. (**c**) Photograph of an all-dielectric metasurface substrate. Six metasurface areas are vertically arrayed. The white scale bar indicates 1 cm. (**d**) Metasurface sensor chip, composed of a self-absorbed pair of an on-top PDMS microfluidic chip with six channels and a metasurface substrate of 45×45 mm${}^{2}$. The chip is set in a holder. (**e**) Actual view of experimental setup around a metasurface sensor chip in the holder. The metasurface sensor chip is connected through tubes. The arrows indicate the direction of liquid flow. The objective lens facilitates access from the top to conduct FL measurement on the metasurface sensor.

Here, we address proof-of-concept experiments for the quantitative detection of COVID-19 spike proteins and Abs. When implementing sandwich assays to detect Ab–protein–Ab complexes, it becomes possible to set the proteins or Abs to be a target in the assay, meaning that two-way detection can be realized in one platform through the employment of all-dielectric metasurface fluorescence (FL) sensors [9,10,11]. The configurations of the two-way assay are schematically illustrated in Figure 1a,b. The optical features of the metasurfaces are described in Section 3.1. Section 3.2 shows that efficient detection of the target glycoprotein can be realized in a practical setup which is superior to the conventional enzyme-linked immunosorbent assays (ELISAs), and that the intrinsic detection capability of metasurface FL sensors reaches sub-pg/mL range in low-background confocal measurements. Additionally, we obtained linear quantitative detection down to 10 picomolars (pM) when setting the Abs as a target; this is elaborated in Section 3.3. Importantly, the all-dielectric metasurface FL sensors were applied to the detection of immunoglobulin G (IgG) Abs in human serums, and it was substantiated that they can retain their high sensitivity even in the presence of abundant other biomolecules, such as albumin, lipids, and other Abs [9]. One of the key factors is binding molecules that are immobilized on the surface of Si nanorods, which specifically capture biomolecules. In this study, we used the same binding molecule used in previous reports [9,10,11]. The metasurfaces were arrayed on a substrate in an assembled manner, as shown in Figure 1c. By combining the metasurface substrate with a microfluidic (MF) chip made of transparent polydimethylsiloxane (PDMS), a metasurface FL sensor was formed (Figure 1d), which is illuminated by the green LED light. The outlook of the experimental setup, including the MF and optical elements, is shown in Figure 1e.

## 2. Materials and Methods

### 2.1. Biosamples

As a testbed for the proof-of-concept experiment, a COVID-19 glycoprotein peptide (ab273063, Abcam, Cambridge, UK) and the corresponding Ab (ab272504, Abcam) were chosen. The glycoprotein peptide with the C terminal was 20 amino acid peptides within the last 50 amino acids of the COVID-19 spike glycoprotein, allowing the rabbit-polyclonal Abs to bind to the spike glycoprotein of COVID-19. The molecular weight (MW) was measured in an electrophoresis setup, and it was confirmed that the Abs had almost the same MW to IgG Abs (Supplementary Materials: Figure S1).

The Abs were originally unconjugated. We labelled the Abs for the sandwich assay, as depicted in Figure 1a,b. 50 μg Ab was used for biotin labeling using a commercial kit (LK03, Dojindo Molecular Technologies, Kumamoto, Japan). Another 50 μg Ab was labeled with HyLite Fluor 555 (HL555) fluorescence molecules using a kit (LK14, Dojindo Molecular Technologies). HL555 has a light absorption peak at 555 nm and emits FL at the wavelength range of 560–660 nm. The HL555-labeling ratio was evaluated by measuring the transmittance of HL555-labeled Abs, which was found to be approximately 7 HL555-molecules/Ab (Supplementary Materials: Figure S2). After labeling, the concentration of HL555-Ab was 0.5 mg/mL in phosphate-buffered saline (PBS, 164-25511, Fujifilm Wako Pure Chemical, Osaka, Japan) of pH 7.4. Biotin- and HL555-labeled Abs were preserved at 5 °C for use in sandwich assay detection. Dilution was conducted each day. The binding of the Abs on Si-nanorod metasurfaces was efficiently conducted using binding molecules of Cys-streptavidin (Cys-SA, PRO1005, ClickBiosystems, Richardson, TX, USA). The detection configurations using the labeled Abs and Cys-SA are shown in Figure 1a,b.

### 2.2. Metasurface Design and Fabrication

**Figure 2:**
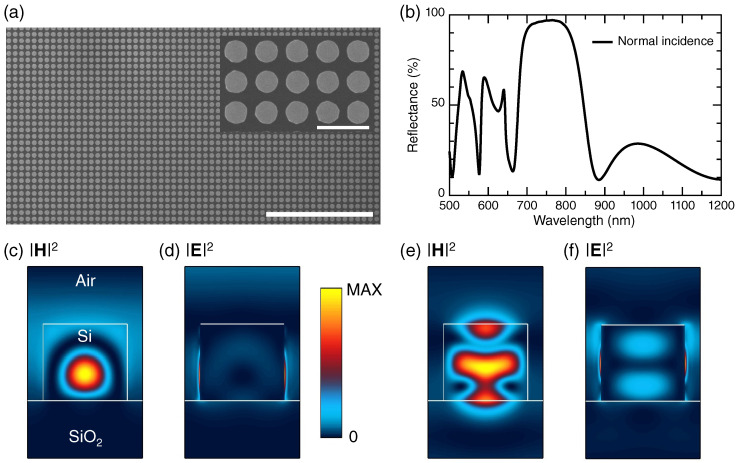
Structure, optical properties, and resonances of all-dielectric metasurfaces. (**a**) SEM image in wide view. The white bold scale bar indicates 5 μm. The inset is shown in a magnified view with a white scale bar of 500 nm as well. The periodicity of the metasurface is 300 nm and diameter of the Si nanorod is 220±3 nm. (**b**) Reflectance spectrum at normal incidence, which was numerically simulated. (**c**,**d**) Respective resonant magnetic and electrical field intensities at 765.1 nm. The vertical section view through the center of the Si nanorod is shown together with the color bar. (**e**,**f**) Respective resonant magnetic and electrical field intensities at 576.5 nm, shown in a similar manner to (**c**,**d**).

The all-dielectric metasurfaces employed in this study (Figure 2a) were conceived by introducing nanostructures in a thin Si waveguide with 200 nm thickness on a SiO${}_{2}$ layer. The silicon-on-insulator (SOI) waveguide is optically isolated, thereby enabling us to study the light-confined modes, which are the source of prominent electromagnetic resonances in all-dielectric metasurfaces [12]. A concrete nanostructure was designed on the basis of the numerical simulations for optical properties using a rigorous coupled-wave analysis [13] and a scattering-matrix algorithm [14]. Figure 2b shows the reflectance spectrum at normal incidence, which was computed for a metasurface of periodicity 300 nm and Si-nanorod-diameter 220 nm; the height of Si nanorod was set to 200 nm, which is equal to the thickness of the SOI layer. Optical resonances appear as reflectance peaks or dips, which were tuned to the HL555-FL-emitting wavelengths of 560–660 nm by adjusting the structural parameters in an empirical manner and through confirmation by numerical simulations. Resonant electromagnetic fields were visualized in the numerical simulation. The resonant fields at 765.1 and 576.5 nm are presented in Figure 2c–f, respectively.

The fabrication of the all-dielectric metasurfaces was conducted via a nanolithography process using electron-beam lithography and dry etching on the SOI wafers. The procedure has already been specified in previous publications [9,10,11]. In this study, the size of metasurface substrates was set to 45×45 mm${}^{2}$ and each substrate had six metasurface areas, as shown in Figure 1c, corresponding to the six MF channels. We note that the metasurface substrates were reusable after washing with an acid solution [15].

### 2.3. MF Protocols

As shown in Figure 1e, sample liquid arrived through tubes, flowed on the metasurface, and exited through a small rotary pump (RP-6R01S-5A-DC3V, Takasago Fluidic Systems, Nagoya, Japan). The MF system is important to ensure the quantitative control of flow rate of liquids in the metasurface sensors. The flow rate was set to 7–80 μL/min, in accordance with the liquid species noted in Section 2.1. The small rotary pump has six channels, and the flow-rate variation between the channels was approximately 5% at designated rates. The PDMS MF chip has six flow channels and forms flow paths, including metasurface areas, when attached to the metasurface substrate in a self-absorbed manner. Each channel has inlet and outlet holes at both ends. The height of the MF flow paths was designed to be 30 μm, and the thickness of the PDMS chip was 2 mm. Liquid samples accessed the MF flow paths through a stainless pin connected to the tubes outside the metasurface sensor (Figure 1e).

In this study, we used the two configurations shown in Figure 1a,b. The target was the glycoprotein peptide in the former configuration, and it was assumed to have diverse target concentrations; indeed, it was diluted to a level less than pg/mL. In the latter configuration, the glycoprotein Ab was the target and varied concentrations, whereas the capture Ab labeled with biotin and the glycoprotein were densely immobilized, flowing at certain concentrations at μg/mL level. Generally, the high-concentration proteins, including Abs, only need a short time (typically, 5 min) of flow at 10–12 μL/min, whereas the low-concentration targets require a low flow rate of 7–10 μL/min for 20 min. Preflow to fill the MF channels with PBS was performed at 70–80 μL/min.

On the basis of these conditions, we conducted the MF protocol for glycoprotein detection on each metasurface as follows: PBS preflow at 400±30 μL, Cys-SA flow 124±4 μL, PBS rinse 273±13 μL, biotin-labeled Ab 142±6 μL, PBS rinse 188±4 μL, glycoprotein peptide 146±6 μL, PBS rinse 221±9 μL, HL555-labeled Ab 134±6 μL, PBS rinse 160±5 μL, and finally a PBS-T (163-24361, Fujifilm Wako Pure Chemical, Osaka, Japan) rinse 275±7 μL. The concentrations of Cys-SA, biotin-labeled Ab, and HL555-labeled Ab were set to 2 μg/mL, 2.5 μg/mL, and 100 ng/mL, respectively. Cys-SA and the labeled Abs were diluted with PBS and diluent NS buffer (ab193972, Abcam), respectively. The target glycoprotein was diluted with PBS.

We refer to the background image that was taken just after the first PBS rinse, and FL images were acquired after the final rinse. The net FL intensities were evaluated by subtracting the background level from the recorded FL images. We mention here that the MF flows required considerable time, approximately 2 h; however, this is shorter than the time needed for conventional ELISA. The MF runtime can be shortened by reducing the rinse time; for example, the PBS rinse just before the PBS-T rinse can be omitted. Total MF time is estimated to be finished within 80 min, and further reduction can be realized to confirm the lowest necessary amount of Cys-SA and labeled Abs in this assay.

Precoating of capture Abs is valid to realize a short-time detection for spike proteins for practical purposes. The biotin-labeled Abs were immobilized in advance and preserved at 5 °C for approximately 20 h. Afterwards, glycoprotein detection was conducted within 1 h. The MF protocol was similar to that noted above.

Ab detection was conducted as follows: PBS preflow 427±7 μL, Cys-SA 126±2 μL, PBS rinse 267±13 μL, biotin-Ab 130±25 μL, PBS rinse 182±5 μL, glycoprotein peptide 146±4 μL, PBS rinse 217±3 μL, HL555-Ab 138±5 μL, PBS rinse 169±3 μL, and a final PBS-T rinse 284±6 μL. In this assay, the target was HL555-labeled Ab flowing at 100, 25, 6.25, 1,56, 0.39, and 0 ng/mL, where the zero concentration means negative control. The concentration of the glycoprotein was fixed at 1 μg/mL, because it played the role of the capture molecule. The concentrations of Cys-SA and the biotin-labeled Ab were the same as those in the glycoprotein detection.

### 2.4. Optical Measurement

FL images of 16-bit depth were acquired using a MF setup equipped with an uncooled CCD camera (Infinity-3S, Teledyne-Lumenera, Ottawa, ON, Canada) which was attached with a 10× objective lens of numerical aperture (NA) 0.28 (M Plan Apo, Mitsutoyo, Kawasaki, Japan). Excitation light came from a green LED (M530F2, Thorlabs, Newton, NJ, USA) and was spectrally reshaped through bandpass filters transmitting a band of 516–538 nm. The green spot (Figure 1d) was introduced with the LED and the objective lens. The FL emitted on the metasurfaces was collected by the objective lens, passed through FL bandpass filters transmitting light of 567–616 nm wavelengths, and was detected by the CCD camera. In the FL measurement, the background level was not as low because the uncooled CCD camera was operated with a gain mode. A typical FL measurement was conducted under conditions of 3 s exposure and 10× gain. Thus, we note that the limit of detection (LOD) was limited by the FL-detection system and not the metasurface sensors.

To examine LOD of the metasurface sensors themselves, an extremely low background FL measurement was conducted in an upright confocal FL microscope (Stellaris 5, Leica Microsystems, Wetzlar, Germany). Low-background measurement was conducted with operating detectors in the photon counting mode. To cover a metasurface area of mm${}^{2}$ dimension, a 5× objective lens of NA 0.15 was used. The excitation wavelength for HL555 was set to 521 nm, the lateral resolution in the confocal microscopy was 0.51×521/NA=1771.4 nm in air, and the detection wavelength was set to a range of 560–620 nm.

## 3. Results

### 3.1. Optical Properties of the Metasurfaces

Figure 2 shows the structural and optical properties of the all-dielectric metasurface. A typical top-view scanning electron microscopy (SEM) image of the metasurface is shown in Figure 2a (a wide view with a scale bar of 5 μm (white bold) and a magnified view (inset) with a scale bar of 500 nm (white)). The periodicity was designed to be 300 nm, and the diameter of Si nanorod was set to 220 nm. The SEM image confirmed that the periodicity is almost exactly 300 nm and the diameter is 220±3 nm.

The optical features of the metasurface can be seen in the reflectance spectrum in Figure 2b. In accordance with the short period length of 300 nm and the 200 nm height of the Si nanorods, the lowest resonant mode in photon energy, which is the longest wavelength mode, appears as a high-reflectance band at 700–800 nm. The lowest mode is ascribed to the magnetic dipole mode because the resonant magnetic field has a single node inside the Si nanorod, as shown in Figure 2c. The electric field is strongly localized at the sidewall of the Si nanorod, as shown in Figure 2d; the distribution is probably suitable for enhancing the electric-dipole transitions, resulting in FL molecules. The magnetic and electric field intensities reach 58.8 and 58.1, respectively, when setting the incident field intensity to 1. Thus, prominent field enhancement is observed on the resonance.

The HL555 molecules have their main FL band at 570 nm. Therefore, it is highly preferable that the metasurface have a resonant magnetic mode around this wavelength. As seen in the reflectance spectrum in Figure 2b, a reflectance dip appears at 576.5 nm. The resonant magnetic and electrical field intensities are shown in Figure 2e,f, respectively. The magnetic field distribution indicates a higher magnetic mode, and the electrical field is strongly localized at the outermost surface of the Si nanorod. The maximum intensities of magnetic and electric fields, when the incident intensities are 1, are 69.8 and 57.5, respectively. This resonance was tuned to enhance the FL emission of the HL555 molecules. It is noteworthy that the total observed enhancement of the FL process was determined by three key factors: excitation efficiency, inner quantum yield in the FL molecule, and FL emission efficiency [16,17,18]. The enhancement of FL emission efficiency is often called the Purcell effect, which describes the expedited rate of electronic transition in resonant electromagnetic fields [19]. The observed enhancement in FL intensity originates from the abovementioned three factors, and the total optimization is essential [20].

### 3.2. Glycoprotein Peptide Detection

**Figure 3:**
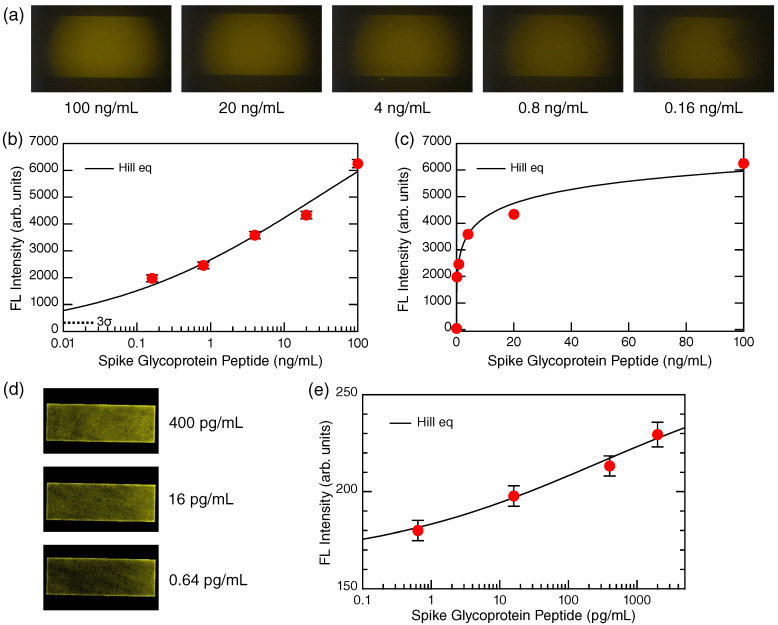
Glycoprotein detection. (**a**) FL images (raw color) of the target concentration from 100 to 0.16 ng/mL. (**b**) Detection data plot in the semi-logarithmic representation. Red dots indicate the FL intensities extracted from the images in (**a**). Error bars are shown together. The solid curve is a fitted curve following the Hill equation (Equation (1)). The level of 3σ from zero is indicated by the dotted line. (**c**) The detection data plot is shown in a linear representation with data in common with (**b**), except for the data point at 0 ng/mL. (**d**) Confocal FL images at low concentrations from 400 to 0.64 pg/mL are shown. The images were originally grayscale, shown by pseudocolor. The colored rectangular areas of 2.1×0.7 mm${}^{2}$ correspond to the metasurfaces. The MF flow was conducted from right to left. (**e**) Detection curve obtained from the confocal images. Note that the abscissa is in units of pg/mL. A curve fitted by the Hill equation (Equation (1)) is shown with the black curve.

Figure 3 shows the spike glycoprotein peptide detection in the configuration illustrated in Figure 1a. The target concentrations were changed from 100 to 0 ng/mL. Figure 3a shows the detected FL images at the nonzero target concentrations, represented with raw color; the brightness was increased by 40% and the contrast was decreased by −40% for better visibility. The FL-emitting areas correspond to the all-dielectric metasurfaces inside the MF channels. Note that although FL-labeled Abs were flowed inside the MF channels, the definite FL was observed only on the metasurfaces owing to their high capability of enhancing FL intensity [18].

The FL intensities extracted from the images in Figure 3a are plotted in Figure 3b,c; the former is a semi-logarithmic representation and the latter is a linear one. The red closed circles denote the FL intensities, which are shown with error bars. The data point at 0 ng/mL can be present in the linear presentation, which is a negative control used to identify the zero level in the measurement. The black curves are fitted detection curves, and are identical to each other in Figure 3b,c, although their appearance is different in the two representations. The detection curve is described by the Hill equation [21]:

(1) $$y=y_{0}+\left(S-y_{0}\right)\frac{x^{n}}{x^{n}+K_{D}^{n}}$$

where *y* denotes the FL intensity, $y_{0}$ is the background level without any target, *S* is the saturation signal intensity, which was regarded as a proportional constant in fitting, *x* is the concentration of target, *n* is the degree of cooperative reaction, and $K_{D}$ is the dissociation constant [22,23]. The parameter *n* suggests an anti-cooperative binding reaction for n<1 and a cooperative reaction for n≥1 [24]. In fitting the data in Figure 3b, the baseline $y_{0}$ was assumed to be 0, while the variables were *n*, $K_{D}$, and *S*. By fitting using commercial graphic software [25], we found that n=0.29, $K_{D}=30.5$ ng/mL, and S=10277. These results suggest that the binding reaction is anti-cooperative, which is often observed between antigens and Abs [9,11]. The Hill equation is mathematically equivalent to the so-called four-parameter logistic equation [9]. From the Hill curve and 3σ level of the dotted line in Figure 3b, where σ is the standard deviation, the LOD was estimated to be 0.8 pg/mL, which is in principle reachable in a very low background measurement.

As described in Section 2.4, low-background FL measurement is generally needed to identify the LOD of the metasurface sensor. Figure 3d shows a set of confocal FL images at the target concentrations of 400, 16, and 0.64 pg/mL. The brightness and contrast of the FL images are set in common. The confocal images are originally grayscale and shown in pseudocolor. The rectangular areas (colored) of 2.1×0.7 mm${}^{2}$ are the metasurface. The MF-flow direction was from right to left; as a result, the right-hand side is relatively bright in all the images, indicating that the immobilization of the biomolecules took place from the right-hand side (or the inlet side of the MF channels). Thus, the confocal FL images provide further insight due to the higher lateral resolution.

The FL intensities in the confocal images were evaluated with settings for analysis at a common analyzing area (Supplementary Materials: Figure S3). The intensities are plotted in Figure 3e as red closed circles with error bars; a fitted curve using the Hill equation (Equation (1)) is shown with a black curve. In the fitting, the parameters *n*, $K_{D}$, and *S* were found to be 0.26, 273.6 pg/mL, and 265.7, respectively. The concentration range in Figure 3e is limited to a low range; therefore, $K_{D}$ and *S* tend to depend on the concentration range when it is narrow, indeed shifting to smaller values than those found in Figure 3b,c. However, *n*, representing the curvature of the fitted Hill curve, is quite close to the parameter *n* in Figure 3b,c, which consistently suggests that the binding reaction of FL-labeled Abs with the target proteins is anti-cooperative. The confocal FL imaging shows that the metasurface sensor is capable of detecting the target even at 0.64 pg/mL, and indicates that the intrinsic LOD is located in the sub-pg/mL range, which is consistent with the estimation using the 3σ line in Figure 3b. In terms of the dynamic range, the metasurface FL sensors were revealed to have a dynamic range more than five orders of the target concentrations, because the FL signals were detected from 100 ng/mL to 0.64 pg/mL in the scaled manner. Although the measured data are a different set from the above, we show confocal FL images at 0.49 and 0 pg/mL (Supplementary Materials: Figure S4). The image at 0 pg/mL indicates the signal level of the negative control. It is shown in the supplementary materials that the FL-signal level at 0.49 pg/mL is located at more than 1σ from that of the negative control while it is within 3σ; therefore, the LOD is located above 0.49 pg/mL, and near 0.64 pg/mL.

**Figure 4:**
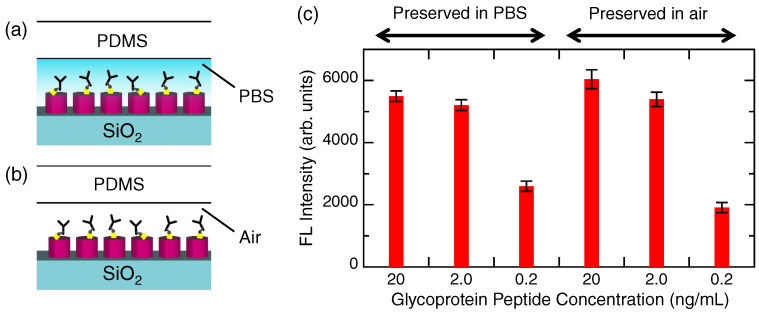
Test for precoating of the capture Abs. (**a**) Precoated Abs were kept in PBS for ∼20 h. (**b**) Precoated Ab was kept in air and dried for ∼20 h. (**c**) After a short runtime of the target glycoprotein peptide and the FL-labeled detection Ab, the detected FL intensities are shown using red bars with error bars in the two cases (**a**,**b**).

Figure 4 shows a series of results on precoating the capture Abs in advance. If the precoating is successful, it becomes possible to substantially reduce detection time in practical situations. Two configurations in the precoating and preservation are illustrated in Figure 4a,b, which have elements in common with Figure 1a. In the former, the capture Abs with the biotin label were preserved in PBS, and in the latter, the capture Ab was dried in air and preserved. The two-type precoated metasurfaces were kept at 5 °C for approximately 20 h.

After preservation, PBS was flowed at 40–45 μL/min for 6 min in all the channels. Subsequently, the glycoprotein, PBS, the HL555-Ab, and PBS-T were flowed within 1 h, in a manner similar to that used for glycoprotein detection in Figure 3. After the flow, the FL images were measured. Figure 4c shows the FL intensities. The target was detected in both cases, indicating that precoating is possible in the assay using the metasurface sensors. We found that preservation in air led to inhomogeneous FL spots; therefore, the recovery process of the dried Ab after preservation should be considered further.

### 3.3. Ab Detection

**Figure 5:**
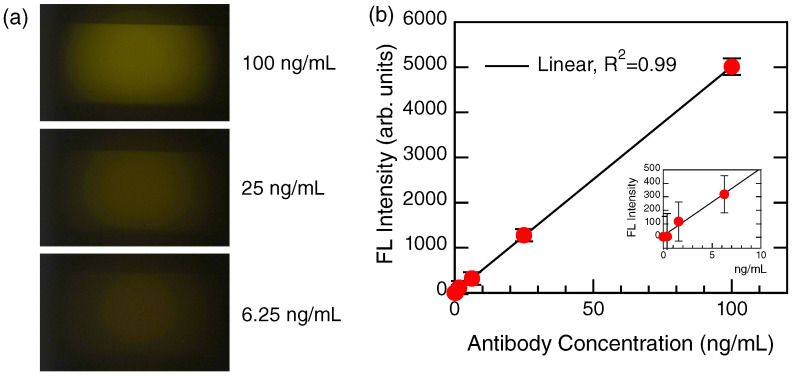
Detection of Abs, as illustrated in Figure 1b. (**a**) FL images (raw color) at different Ab concentrations from 100 to 6.25 ng/mL (or 686 to 41.2 pM). (**b**) Linear plot of the FL intensity for the Ab concentrations. The measured data are shown with red closed circles with error bars. The black line is linearly fitted. The inset magnifies a low-concentration range.

Figure 5 shows a set of experimental results from the Ab detection illustrated in Figure 1b. The glycoprotein was densely immobilized on the metasurface sensor, and the Abs at different concentrations were detected. In this proof-of-concept experiment, the target Abs were labeled with HL555, and direct detection of the Abs was conducted on this basis.

Three FL images at different Ab concentrations from 100 to 6.25 ng/mL are shown in Figure 5a; the representation setting is similar to that in Figure 3c. The detection profile is shown in Figure 5b. The FL intensities (red closed circles) were evaluated from the measured FL images, shown with error bars. Obviously, the profile is almost linear, with a variance of $R^{2}=0.99$. The inset magnifies a low-concentration range and shows that the linear response holds to 0 ng/mL. Thus, the linear response is a good feature to provide a quantitative assay for the Abs.

## 4. Discussion

### 4.1. Glycoprotein Peptide Detection

It was demonstrated that the metasurface FL sensor is capable of a very low target concentration of 0.64 pg/mL. We employed confocal FL microscopy to perform extremely low background measurement, observing that even the low concentrations could be detected in a scaled manner, as shown in Figure 3e. This suggests that the LOD of the metasurface FL sensor itself exists at a lower concentration than the measured concentration of 0.64 pg/mL.

In a practical setup using an uncooled CCD camera, the target glycoprotein peptide was detected down to approximately 0.1 ng/mL. The detection range was limited by the performance of the CCD camera. However, this detection range is better than that of conventional ELISA, which shows an LOD of 0.5 ng/mL [26]; the dynamic range was estimated to be 1–64 ng/mL from open data. In terms of detection runtime, the present method is substantially faster than conventional ELISAs, which usually take at least several hours.

Table 1 lists detection performance on the several platforms addressed in this article. Here, we define dynamic range as a range scaled by a rational equation and distinguish signals between one-order different target concentrations in the 1σ criterion. Runtime means all the time that is needed for a test, and is inseparable.

### 4.2. Ab Detection

In Ab detection, the IgG-type Ab was detected even at 1.56 ng/mL, which is 10.7 pM, in a linear manner. Table 1 lists the Ab detection results. IgG-type COVID-19 Ab was detected at a range of 5–100 nanomolars (nM) in a previous report using gold nanoparticles [27], where the linear dynamic range was limited to a range from 100 nM to approximately 10 nM, which was claimed to meet the detection of neutralizing Abs. Thus, the present metasurface sensors exhibit approximately 100-fold better detection performance, thereby being sufficient for qualifying the neutralizing Abs.

Next, we discuss the robustness of the all-dielectric metasurface sensors for impeding biomolecules. It has been reported that Ab detection in metasurface FL sensors is robust when human serum is included in the target buffer [9]. As is widely known, human serums contain abundant biomolecules such as albumin, lipin, IgG, and more; for example, IgG is typically included at 87–170 mg/mL. In the previous publication [9], it was demonstrated that the target IgG at 10 ng/mL can be detected without substantial loss. Thus, even when the impeding IgG is more than 100,000-fold more dense than the target IgG, all-dielectric metasurface sensors can detect the target. Although we focus here on demonstrating the detection of glycoproteins and Abs, we believe that the metasurface sensors retain this robustness.

For optical detection of proteins such as Abs, resonant wavelength-shift measurements are often reported. Regarding COVID-19 IgG, a very low LOD of 30 aM has been reported [28]; such low LODs have sometimes been claimed for other biomolecules as well [29]. However, the amount of wavelength shift for the COVID-19 IgG is <10 nm for eight-order concentration changes. As a result, similar concentrations are often indistinguishable; for example, the experimental data indicate that the method cannot discriminate 1 nM from 0.1 nM or 0.1 nM from 0.01 nM [28]. Thus, the technique has a disadvantage in quantifying the target IgG concentrations even at sub-nM ranges as compared with the present metasurface FL sensors. Resonant-shift assays stemming based on surface plasmon resonance [30] have been studied for more than thirty years and have yet to be established as a high-sensitivity method, likely for this very reason.

### 4.3. Further Designs for Metasurface FL Sensors

There are many reports on non-empirical designs for metasurfaces [31,32,33,34,35]. Nevertheless, trials for FL-enhancing metasurfaces have hardly been reported. This is partially because FL enhancement involves excited electronic states in molecules, and simulations for the metasurface design cannot handle the excited states. To implement simulations including excited-state dynamics, it is necessary to incorporate light–matter interaction. Thus, designs for metasurface FL sensors are generally difficult. However, in Section 3.1 we have described features in electromagnetic resonance that are suitable for efficient FL enhancement; therefore, we consider that clues to finding other structures suitable for FL sensing are provided by the features in the resonant modes.

## 5. Conclusions

In this study, we performed a series of proof-of-concept experiments to detect COVID-19 spike glycoproteins and Abs in one platform, i.e., an all-dielectric metasurface FL sensor. In a practical setup, the glycoprotein was detected more efficiently than when using conventional ELISA. In addition, the LOD of the metasurface sensors was tested using confocal microscopy; the LOD was found to be <0.64 pg/mL, and the dynamic range was more than five orders of the target concentrations. Thus, the metasurface FL sensors have extremely high-sensitivity. Moreover, the Ab detection was linear down to ∼10 pM, which, to our knowledge, is highly reliable in terms of quantification ability. The Ab detection range is sufficient for testing the neutralizing Abs. As discussed in Section 4.2, all-dielectric metasurface sensors are robust for impeding biomolecules. Overall, the present method is highly promising for straightforward extension to practical situations.

## Figures and Tables

**Table 1:** COVID-19 detection for proteins and antibody. ICH denotes immunochromatography-based disposable test kits. NA denotes not available. Dynamic range defined in the text is estimated from the published data in the references. Metasurface sensor refers to this study. Runtime includes all the preparation time that is inseparable in a test.

Target	Method	Dynamic Range	Runtime
Spike proteins	ICH	NA	20–30 min
Spike glycoproteins	ELISA [26]	1–64 ng/mL	4–6 h
Spike glycoproteins	Metasurface sensor	0.64–100,000 pg/mL	<1 h
Glycoprotein Ab	Metasurface sensor	1.56–100 ng/mL	30 min
(IgG-type)		(or 10.7–686 pM)	
IgG	Resonant shift [27]	5–100 nM	70 min
IgG	Resonant shift [28]	1–10 nM	overnight

## Data Availability

Data in this article are available from the corresponding author upon reasonable request.

## Supplementary Materials

The following are available online at https://www.mdpi.com/article/10.3390/bios12110981/s1, Figure S1: molecular weight analysis, Figure S2: transmission spectra of HL555-labeled Ab, Figure S3: FL-image analysis with setting an analyzing box, Figure S4: confocal FL images including a negative control. References [36,37,38] are cited in the supplementary materials.
