# Supplementary material for: Two-Way Detection of COVID-19 Spike Protein and Antibody Using All-Dielectric Metasurface Fluorescence Sensors
Source: Biosensors (Basel). 2022 Nov 7;12(11):981. doi: 10.3390/bios12110981 (PMC9688339; doi:10.3390/bios12110981)
Supplement: Supplementary file 1 [file biosensors-12-00981-s001.zip › biosensors-1967694-Supplmentary.pdf]

Supplementary Materials

Two-Way Detection of COVID-19 Spike Protein and Antibody Using All-Dielectric Metasurface Fluorescence Sensors

Masanobu Iwanaga and Wanida Tangkawsakul

Research Center for Functional Materials, National Institute for Materials Science (NIMS),

1-1. Namiki, Tsukuba 305-0044, Japan

S1. Molecular weight analysis

The electrophoresis [1] was carried out for anti-SARS-CoV-2 spike glycoprotein antibody (ab272504, Abcam, UK) were separated on a 15% gel (E-R15L, Atto, Japan). The unconjugated antibody, HiLyte Fluor 555 (HL555)-labeled antibody, biotin-labeled antibody and Cys-streptavidin (Cys-SA) were mixed to sample buffer (EzApply, AE-1430, Atto) in ratio 1:1. Each 60 μg sample was added at 18 μL in a well. The proteins were driven at 21 mA for 120 min. Molecular weight (MW) standard was Novex Sharp Pre-stained Protein Standard (LC5800, Invitrogen). The gel was stained with Coomassie Brilliant Blue Stain Kit (30035-14, Nacalai Tesque, Japan).

Comparing to the MW standard, the MW for each sample was determined. The MW of the antibody shows 160,000 and higher values, which were slightly larger than theoretical IgG MW of 150000. The biotin-labeled and fluorescence (FL) molecule HL555-labeled antibodies show broader MW bands than the band of unconjugated antibody, suggesting larger MWs. Cys-SA band indicates tetramer formation because the MW band appears at approximately 60,000 and the MW of the monomer streptavidin is 15000.

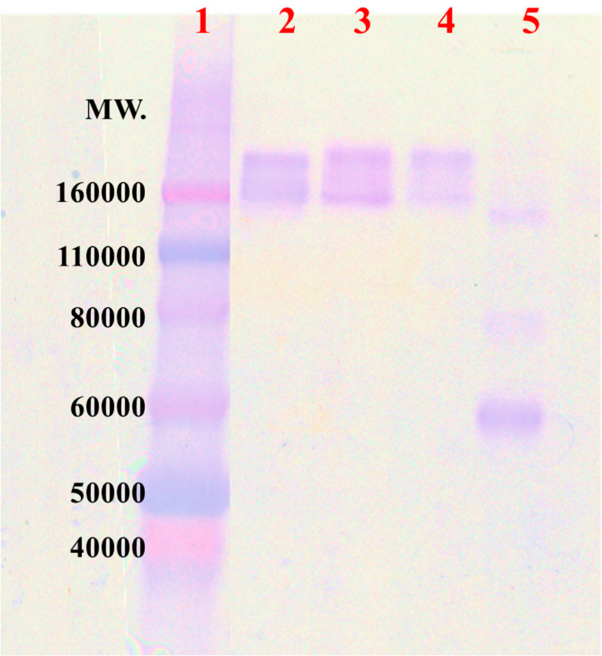

Figure S1. Electrophoresis Result. Lane 1 was loaded with a standard protein marker. Lanes 2-4 were loaded with unconjugated antibody, HL555-antibody, and biotin-antibody, respectively. Lane 6 was loaded with Cys-SA.

S2. Labeling ratio of fl molecules on antibody

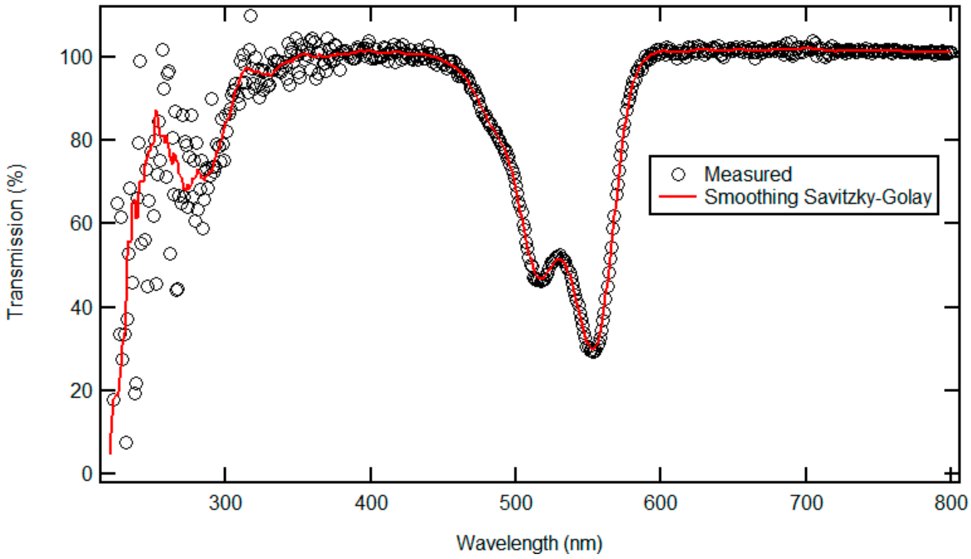

Figure S2. Measured transmittance of HL555-labeled antibody (black open circles). Red curve is a smoothed curve of the measured data.

The anti-SARS was labeled using HL555 Labeling Kit-NH2 (LK14, Dojindo Molecular Technologies, Japan). Labeling ratio of HL555 to the antibody can be evaluated by measuring light absorbance of the protein solution at 280 nm and 555 nm. Figure S2 shows measured transmission data (black open circles) of HL555-antibody PBS solution of 0.1 mg/mL and the smoothed curve (red line) fitted by the 31th-order Savitzky-Golay algorithm.

The ratio HL555/anti-SARS is given by the following equation [2]:

$$\boldsymbol{Ratio\ (HL555: antibody)} = \frac{\boldsymbol{A_{555}/150000}}{\boldsymbol{(A_{280} - A_{555} \times 0.1)/(\varepsilon\ of\ antibody)}}$$

The antibody is IgG-type. We substitute 216,000 for ε. The molar absorption coefficient of HL555 is 150,000. A_{555} is absorbance at 555. A_{280} is absorbance at 280. From the smoothed data in Figure S2, we evaluated A_{555} = 0.51 and A_{280} = 0.15, and finally found that the ratio of HL555 to the antibody is 7.4:1, which means that 7.4 HL555 molecules bind on an antibody in average.

S3. fl image analysis

From FL images, the FL intensity was analyzed. Usually, FL intensity in the image is evaluated by making histogram. We used an image-analysis software, ImageJ [3]. Choosing an appropriate area in a FL image, histogram is evaluated; normally, one peak appears and the mean corresponds to averaged FL intensity and the peak width is the standard deviation of the histogram. Figure S3 shows a box setting in a confocal FL image; the box was commonly set in the series of confocal images; the resultant values are plotted in Figure 3e. Note that Figure S3 is shown in the original gray scale. Rectangular light gray area is the all-dielectric metasurface. Inside the analyzing box (yellow), averaging was executed to reduce the standard deviation coming from the instrument. In ImageJ, an smoothing option, named Gaussian Blur, was implemented.

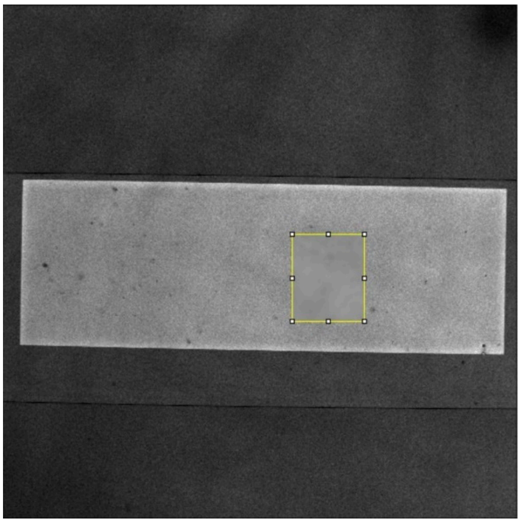

Figure S3. FL image with an analyzing box (yellow). The box is commonly set to the series of the confocal FL images. In the box, an averaging algorithm was executed to reduce the data fluctuation coming from the instrument.

S4. confocal FL images

In Figure S4, we provide confocal FL images, which are supplementary to Figure 3d. Figure S4a shows a confocal FL image of detected amplicons generated from the target glycoprotein concentration of 0.49 pg/mL; the intensity histogram is shown in Figure S4b. The histogram was evaluated going through a smoothing filter, Gaussian Blur 2%, in ImageJ [3]. The mean of the histogram was 69.482 and the standard deviation (σ) was 3.708. Figure S4c shows a confocal image measured for the negative control, *i.e.*, the 0 g/mL target. In Figure S4d, the mean of the histogram was 64.774 and the standard deviation was 4.255. Thus, the mean at 0.49 pg/mL is out of 1σ from the negative control; however, the former is within 3σ from the latter. In a normal statistical viewpoint, we consider that the limit of detection exists at a higher concentration than 0.49 pg/mL, which is consistent with the conclusion in the text.

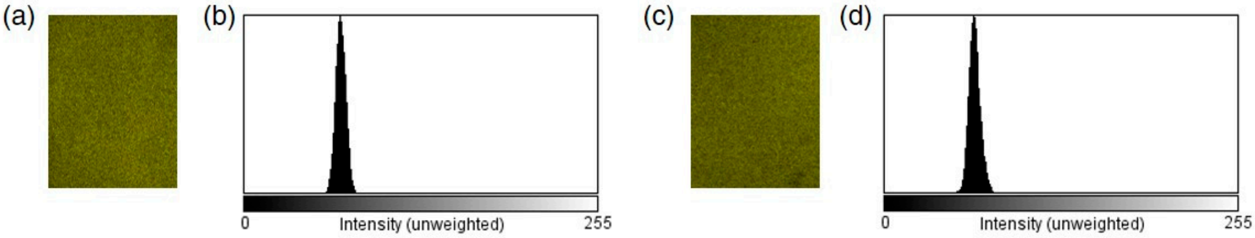

Figure S4. Confocal FL images and intensity histograms. (a) Confocal FL image at the target concentration of 0.49 pg/mL. (b) Intensity histogram corresponding to (a). (c) Confocal FL image of negative control (*i.e.*, 0 g/mL). (d) Intensity histogram corresponding to (c). The colors in (a) and (c) are shown with a pseudo-color; the original images were gray scale like Figure S3.

References

[1] Laemmli, U. K. (1970). Cleavage of structural proteins during the assembly of the head of bacteriophage T4. nature, 227(5259), 680-685.

[2] Manual for FL Labeling Kit. https://www.dojindo.co.jp/manual/LK14e.pdf (accessed September 21, 2022).

[3] ImageJ homepage at https://imagej.nih.gov/ij/ (accessed September 23, 2022).
